# Facile One-Pot Synthesis of Bimetallic Co/Mn-MOFs@Rice Husks, and its Carbonization for Supercapacitor Electrodes

**DOI:** 10.1038/s41598-019-45169-0

**Published:** 2019-06-20

**Authors:** Hyunuk Kim, Muhammad Sohail, Chenbo Wang, Martin Rosillo-Lopez, Kangkyun Baek, Jaehyoung Koo, Myung Won Seo, Seyoung Kim, John S. Foord, Seong Ok Han

**Affiliations:** 1 Energy Materials Laboratory, Korea Institute of Energy Research, 152 Gajeong-ro, Yuseong-gu, Daejeon, 34129 Republic of Korea; 2 Green Fuel Laboratory, Korea Institute of Energy Research, 152 Gajeong-ro, Yuseong-gu, Daejeon, 34129 Republic of Korea; 3 Advanced Energy and System Technology, University of Science and Technology (UST), Daejeon, 34113 Republic of Korea; 4 Center Center for Self-assembly and Complexity, Institute for Basic Science, 77 Cheongam-ro, Nam-gu Pohang, 37673 Republic of Korea; 5 Department of Chemistry, University of Oxford, South Parks Road, Oxford, OX1 3TA United Kingdom

**Keywords:** Composites, Porous materials, Supercapacitors, Metal-organic frameworks

## Abstract

Novel hybrid nanomaterials comprising metal-organic framework compounds carbonised in the presence of biomass material derived from rice husk have been investigated as a new class of sustainable supercapacitor materials for electrochemical energy storage. Specifically, two synthetic routes were employed to grow Co/Mn metal-organic framework compounds in the channels of rice husks, which had been activated previously by heat treatment in air at 400 °C to produce a highly porous network. Pyrolysis of these hybrid materials under nitrogen at 700 °C for 6 h produced metal-containing phases within the nanocarbon, comprising intimate mixtures of Co, MnO and CoMn_2_O_4_. The materials thus produced are characterized in detail using a range of physical methods including XRD, electron microscopy and X-ray photoelectron spectroscopy. The synthetic pathway to the metal-organic framework compound is shown to influence significantly the physical properties of the resulting material. Electrochemical evaluation of the materials fabricated revealed that higher specific capacitances were obtained when smaller crystallite sized bimetallic Co/Mn-MOFs were grown inside the rice husks channels compared to larger crystallite sizes. This was in-part due to increased metal oxide loading into the rice husk owing to the smaller crystallite size as well as the increased pseudocapacitance exhibited by the smaller crystallite sizes and increased porosity.

## Introduction

Supercapacitors store charge *via* the electric double layer (EDL) and have attracted a lot of attention because of their high power density, fast charging time, low maintenance cost and long cycle life^1,2^. In general, their capacitance is proportional to the surface area of the conductive electrodes. Hence porous carbon with large surface area has been conventionally used as a cheap and effective electrode material^3–7^. However, improvement in performance is a long term objective and is the development of more sustainable manufacturing conditions. Novel pseudocapacitive nanomaterials which store energy electrochemically through surface redox reactions as well as the EDL have been extensively investigated as an electrode to increase both power and energy density^8–11^. To date, as a result, transition metal oxides, conducting polymers and heteroatom doping carbonaceous materials have been proposed as pseudocapacitive electrode materials^12–19^. Transition metal oxides among various pseudocapacitive nanomaterials, have shown potential as an electrode material because of their high theoretical capacitance, low cost and reversibility. In particular, cheap hybrid electrodes such as MnO_2_/carbon and CoO/carbon exhibited fast and stable redox reactions^20–29^.

Metal-organic frameworks (MOFs) assembled from metal ions and organic ligands have well-ordered nanostructures with high surface area and functionality^30^. The secondary building units representing the point of extension in MOFs is mostly composed of metal oxide clusters^31,32^. Therefore, the carbonization of MOFs under inert atmosphere generates metal oxides in carbonaceous materials^33^. These materials have a large surface area with well dispersed metal oxides within the carbon framework. Consequently, MOF-derived metal oxide/carbon has shown promising platform applications in clean energy^34,35^. For example, Mn-MOF-derived Mn_2_O_3_/graphene showed a high specific capacitance of 471 Fg^−1^ in 0.5 M Na_2_SO_4_ and long-cycle stability^36^. Interestingly, the pyrolysis of MOFs under air or oxygen atmosphere produced well-defined metal oxides with enhanced pseudocapacitive properties. For example, mixed-metallic Co_3_O_4_/NiCo_2_O_4_ prepared by the pyrolysis of ZIF-67/Ni-Co LDH under air exhibited a high specific capacitance of 972 Fg^−1^ at current density of 5Ag^−1^ ^37^.

In this work, two different-sized bimetallic Co/Mn-MOFs were grown in the channels of pre-activated rice husks (RHs) and pyrolyzed to produce Co/MnO/CoMn_2_O_4_@RHs for pseudocapacitive electrodes. RH is one of the cheapest and most abundant agricultural organic wastes obtained from the milling of rice, hence can be considered as a highly sustainable nanocarbon material for technological applications. Indeed, along with sugarcane bagasses, it is one of the two largest-volume agricultural wastes^38^. More than 120 million tons of RHs per year are produced over the world, but the demand for applications is at present very low^39^. It has been only used in limited areas, for instance as a fertilizer, fuel, insulating and building material, landfilling or paving material^40,41^. Unlike other biomass-derived nanocarbons such as seaweeds, cherry stones and coffee shells^42–45^, one of the most interesting aspects of RHs is that it has rigid and thick-walled micrometer-scale channels after pre-activation at 400 °C, which can work as a conductive host.

**Figure 1:**
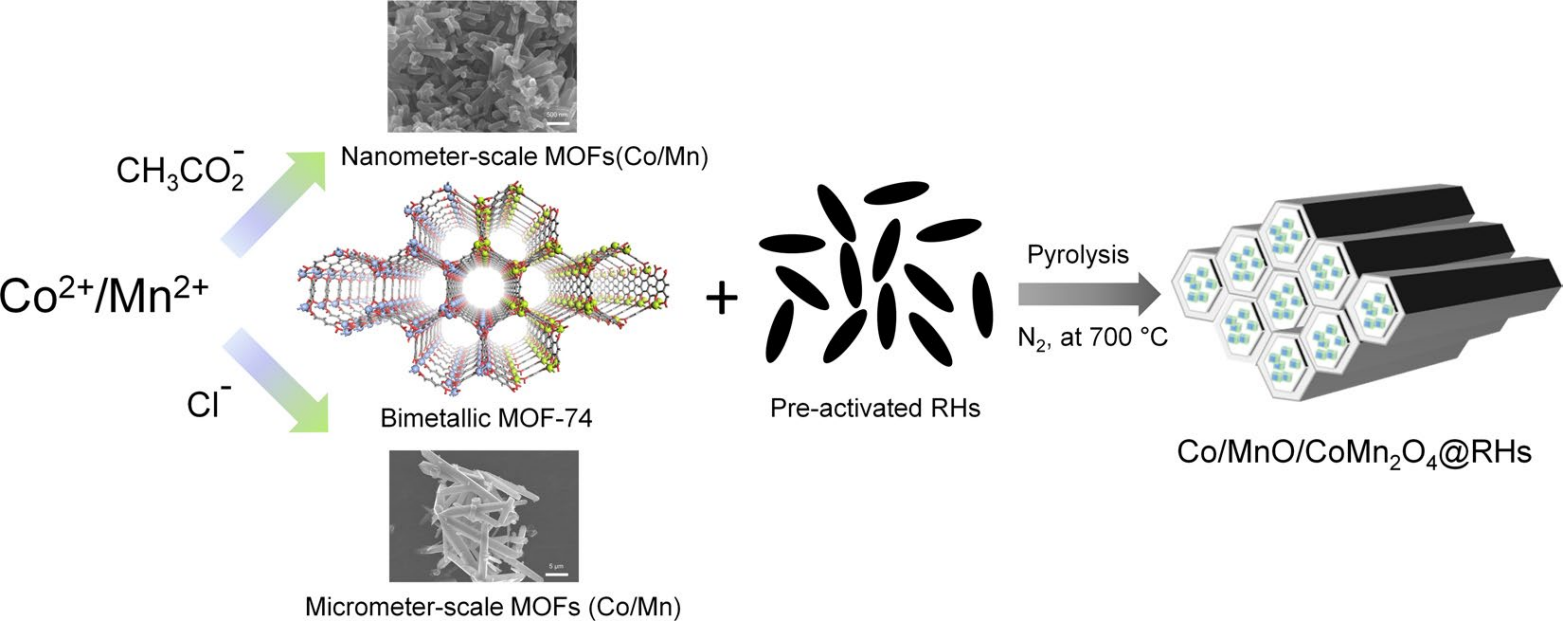
The schematic illustration of the synthesis of Co/MnO/CoMn_2_O_4_@RHs derived from MOF@RHs.

Herein we demonstrate for the first time the *in-situ* growth of two different crystallite sized bimetallic Co/Mn-MOFs inside the channels of pre-activated RHs and relate the pseudocapacitive performance of the hybrid materials to the crystallite size (Fig. 1). In particular, the work establishes for the first time that hybrid nanocarbon materials from MOFs and biomass derived nanocarbons, as a sustainable form of carbon can be used for a new approach to supercapacitor devices. In addition the work provides interesting insights into how the synthesis conditions for the MOF influences significantly the device performance obtained.

## Results and Discussion

### *In-Situ* Crystallization of Bimetallic MOFs in RHs

**Figure 2:**
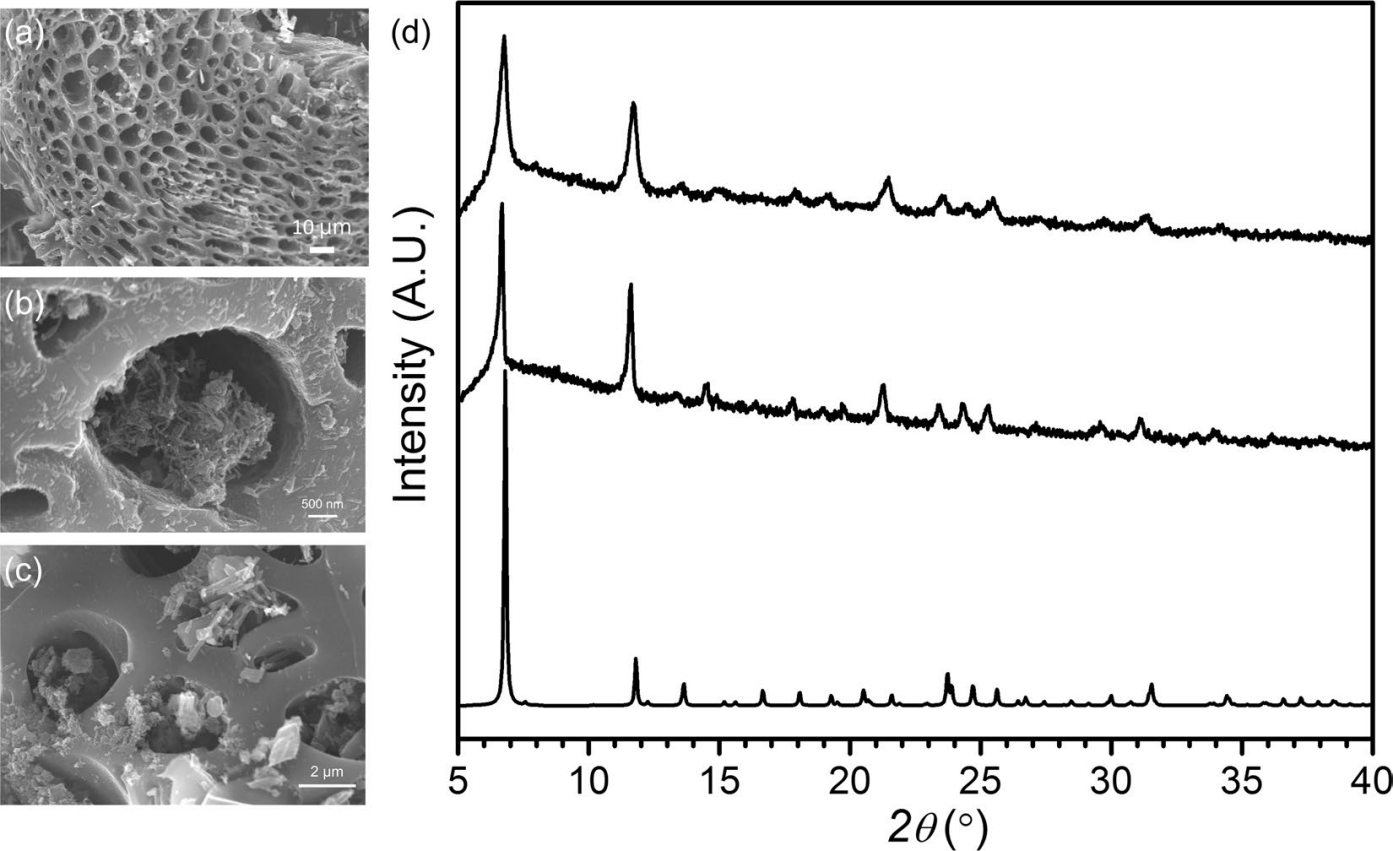
(**a**) SEM images of (**a**) carbonized RHs at 400 °C and (**b**) **1**@RHs, (**c**) **2**@RHs and (**d**) Measured powder XRD profiles of **1**@RHs (top), **2**@RHs (middle) and calculated one for MOF-74 (bottom).

Phase-pure MOF-74 with mixed Mn(II) and Co(II) were prepared by a one-pot solvothermal reaction in the presence of two different precursors. SEM-EDS revealed that **1** and **2** have both Mn(II) and Co(II) in a single crystal with Mn/Co ratios of 0.94 and 1.02 respectively (Fig. S1). The bimetallic crystalline **1** and **2** showed the same PXRD patterns as one single-phase, which is consistent with the calculated PXRD profile of MOF-74 (Fig. S2). The Halder-Wagner method revealed that the crystallite size (6.47 nm) of **1** is almost eight times smaller than that (47.2 nm) of **2** (Fig. S3). Such crystallite sizes of bimetallic MOFs influence the growth of crystals; **1** has hexagonal rod shapes with lateral dimensions of several hundred nanometers, while **2** has similar rod shapes with dimensions on the order of several microns. The different crystal sizes of **1** and **2** can be explained by considering the nucleophilicity of the counter anions to the metal cations. Acetate during the crystallization of **1** is less solvated than chloride during that of **2** in polar and aprotic solvents such as DMF, which is a major solvent, indicating a higher nucleophilic reactivity of acetate to Co^2+^ and Mn^2+^ ions^46,47^. Therefore, metal complexes with acetate are slow to dissociate and participate in the crystallization of **1**, and hence generates smaller crystals than that of chloride. To incorporate bimetallic MOFs in RHs, **1** and **2** were *in-situ* crystallized in the presence of pre-activated RHs at 400 °C. SEM-EDS shows that **1**@RH and **2**@RH have Mn/Co ratios of 0.86 and 0.87. PXRD analysis of **1**@RHs and **2**@RHs shows that both samples have an identical phase to those of **1** and **2**. SEM analysis reveals that **1** and **2** were grown in the micrometer pore size channels of RHs (Figs 2, S4). As observed by SEM analysis, the crystallite size of **1** in RHs is much smaller than that of **2** in RHs, consistent with the observations relating to pure **1** and **2** as discussed above (Fig. S5). TGA analysis of **1**@RHs and **2**@RHs revealed that the loading amount of **1** and **2** in the channels of RHs is 25.3% and 17.3%, respectively (Fig. S6).

### Carbonization of MOF@RHs and their Electrochemical Properties

**Figure 3:**
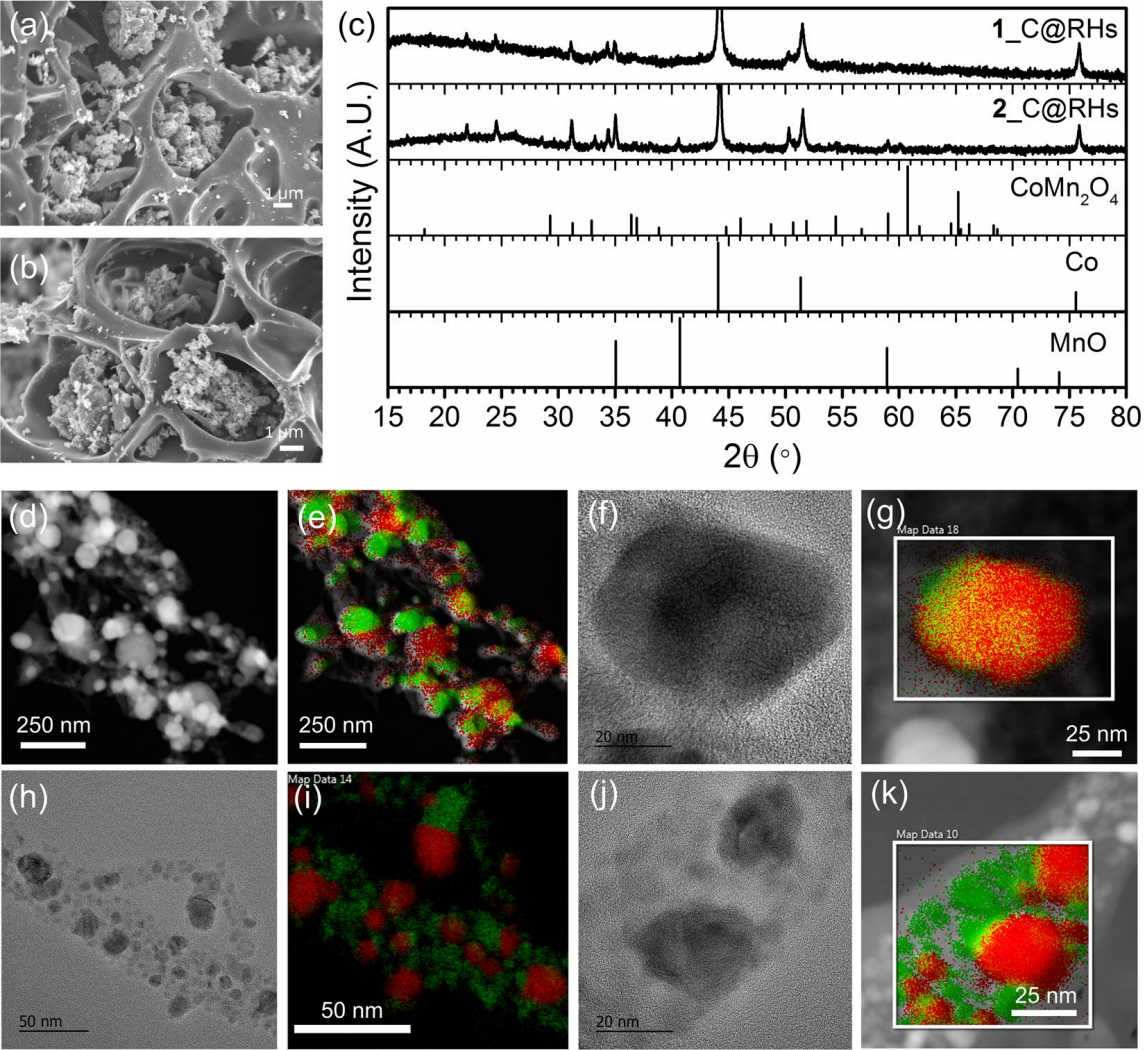
SEM, STEM, EDS mapping, HRTEM images of **1**_C@RHs (**a**,**d**–**g**) and **2**_C@RHs (**b**,**h**–**k**) (green: Mn, red: Co), and (**c**) XRD patterns.

To synthesize conductive pseudocapacitive materials derived from MOFs, the **1**@RHs and **2**@RHs samples were pyrolyzed under N_2_ atmosphere at 700 °C for 6 hours. SEM images of **1**_C@RHs and **2**_C@RHs show that crystalline materials were still located in the microchannels of RHs (Fig. 3a,b). PXRD analysis confirms that bimetallic **1** and **2** are completely decomposed and changed their crystalline phase (Fig. 3c). Rietveld refinement of PXRD profiles reveals that **1**_C@RHs and **2**_C@RHs have three crystalline phases corresponding to cubic Co, cubic MnO and tetragonal CoMn_2_O_4_. (Fig. S7). The peaks appearing at 44.2°, 51.5° and 75.9° are ascribed to the (111), (200), and (220) reflections of cubic Co (JCPDS card No. 15–0806). The diffraction peaks observed at 34.9°, 40.6°, 58.8°, 70.3° and 73.8° are assigned to the (111), (200), (220), (311), and (222) reflections of cubic MnO (JCPDS card No. 07–0230). The remaining peaks at 31.5°, 33.2° and 50.3° correspond to (200), (103) and (204) reflections of tetrahedral CoMn_2_O_4_ respectively (JCPDS card No. 77–471). STEM-EDS and HRTEM analysis also show that most of nanoparticles are composed of either Co or Mn, but some of them contain Co and Mn together, suggesting the formation of bimetallic oxides in single particles (Fig. 3b–e,g–j). Interestingly, a close inspection of HRTEM images reveals that some of single particles are embedded in the graphitic carbons, which is due to the catalytic graphitization process by Co species (Fig. S8)^48^. This graphitic carbon on Co species can enhance the electric conduction for supercapacitor electrode.

**Figure 4:**
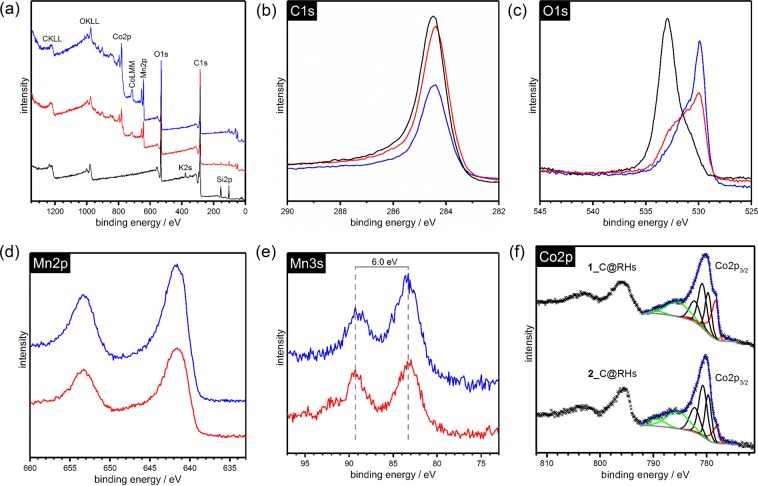
XPS spectra of RHs (black), **1**_C@RHs (blue) and **2**_C@RHs (red): (**a**) survey spectra, (**b**) high-resolution C1s region, (**c**) O1s region, (**d**) Mn2p region, (**e**) Mn3p region and (**f**) deconvolution of the Co2p_3/2_ region.

XPS survey spectra of RHs, **1**_C@RHs and **2**_C@RHs are shown in Fig. 4(a). Analysis of the spectra revealed that carbon and oxygen were present in all three samples, as expected, however only potassium was found in the unmodified RHs. A large amount of silicon (~10%) was also found to be present in the RHs, although none was detected in the **1**_C@RHs, and only a trace amount was observed in the **2**_C@RHs. Both **1**_C@RHs and **2**_C@RHs contained manganese and cobalt although 2–3 times more of the bimetallic species was seen in the **1**_C@RHs compared to **2**_C@RHs, *c*.*f*. Table 1. The values in Table 1 reflect the mean and sample standard deviation (1.s.d.) of the metal/carbon (M/C) ratios observed in the XPS survey spectra of **1**_C@RHs and **2**_C@RHs respectively. In each case, three 400 µm spot sizes were used to generate the relevant statistics. The small standard deviation in each sample illustrates the homogeneity of the metal dispersion throughout the carbon framework. Interestingly, the Mn:Co ratio was 1:1 as expected for the **1**_C@RHs, although this was not the case for the **2**_C@RHs which exhibited a higher loading of manganese compared to cobalt (Mn/Co = 1.43).

**Table 1:** Atomic ratios of RHs, **1**_C@RHs and **2**_C@RHs according to XPS survey spectra.

Sample	Mn/C	Co/C	(Mn + Co)/C
**1_C**@RHs	0.124 ± 0.004	0.119 ± 0.009	0.24 ± 0.01
**2_C**@RHs	0.053 ± 0.001	0.037 ± 0.004	0.090 ± 0.006

Interpretation of the XPS high resolution regions reveals a C1s peak at 284.5 eV for all samples, which reflects the graphitic nature of the samples. The O1s peak centered at 532.9 eV for the RH is ascribed to SiO_2_, while the peaks centered at 529.9 eV for **1**_C@RHs and **2**_C@RHs are attributed to the metal oxides^49–51^. In each case, there will also likely be contributions from metal hydroxide, hydrated or defective oxides (on the surface) at ~530 eV as well as C–O/C=O organic species within the 531–533 eV region^52,53^. Similarly, a contribution from water at ~533.5 eV is also assumed. Note the presence of metal carbonates is not expected as these were absent in vthe PXRD patterns of **1**_C@RHs and **2**_C@RHs. In the Mn3s region, **1**_C@RHs and **2**_C@RHs show a peak splitting separation of 6.0 eV indicating MnO species, which is consistent with the PXRD data^54,55^. However, the possibility of Mn(III) species such as Mn_2_O_3_ cannot be ruled out, owing to the low signal to noise ratio. Similarly, the Mn2p region shows characteristic peaks for the presence of manganese oxides (Mn2p_3/2_ and Mn2p_1/2_ are broad and centered at 641.8 and 653.0 eV respectively)^53^. Deconvolution of the Co2p_3/2_ region in Fig. 4(f) reveals a shoulder feature at ~778.3 eV (the most pronounced red peak) which can be unequivocally assigned to Co metal in both the **1**_C@RHs and **2**_C@RHs^53^. Similarly, a small contribution from the Co LMM Auger can also be seen at lower binding energy of the Co metal peak (fitted peak in orange). The remaining fitted peaks in black and green can be assigned to CoMn_2_O_4_ and their satellites respectively. Typically, Co(II) peaks come at a higher binding energy than Co(III), whilst the converse is true of the satellite features^53,56^. The fitting of the Co2p_3/2_ region was carried out as described by Biesinger and coworkers^53^. The mixed bimetallic CoMn_2_O_4_ species was fitted by considering the fitting parameters used for Co_3_O_4_ which is also a mixture of Co(II) and Co(III) cations. However, the area was not constrained as the ratio of Co(II) and Co(III) ions is unknown. Lastly, a background offset for the deconvolution was found to not improve the peak fitting of the Co2p_3/2_ region.

**Figure 5:**
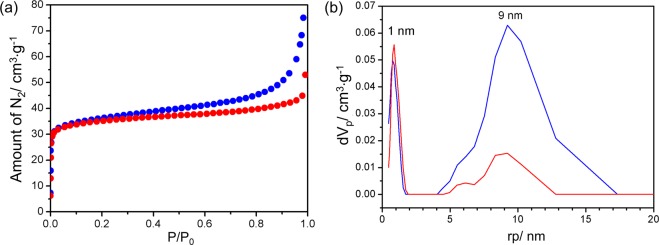
(**a**) N_2_ adsorption isotherms and (**b**) PSDs of **1**_C@RHs (blue) and **2**_C@RHs (red).

N_2_ adsorption measurements at 77 K for **1**_C@RHs and **2**_C@RHs shown in Fig. 5 were carried out to investigate the BET surface area and porous properties of these samples (Table 2) The N_2_ adsorption isotherms show a quick saturation at low pressure followed by slow increase at high pressure indicating the coexistence of micropores and mesopores. The pore size distributions (PSDs) estimated by Non-Local Density Functional Theory (NLDFT) show two characteristic pores at 1 nm and 9 nm. The mesopore volume of **1**_C@RHs at 9 nm is much higher than that of **2**_C@RHs, despite the fact that the surface areas and micropore volumes are almost same. Consequently, it is expected that a higher mesopore volume will result in an increase in overall capacitance due to the diffusion of electrolyte ions into the pores.

**Table 2:** Porosities and BET surface of the samples.

Samples	BET SA (m^2^/g)	Total pore volume (cm^3^/g) at P/P_0_ = 0.99	Micropore volume^a^ (cm^3^/g)	Mesopore volume^b^ (cm^3^/g)
RHs^c^	121	0.2824	0.2019	0.0805
**1**_C@RHs	136	0.1160	0.0386	0.0792
**2**_C@RHs	137	0.0808	0.0411	0.0397

^a^Determined by t-plot analysis.

^b^Mesopore volume = total pore volume – micropore volume.

^c^See Fig. S9 in Supporting Information.

**Figure 6:**
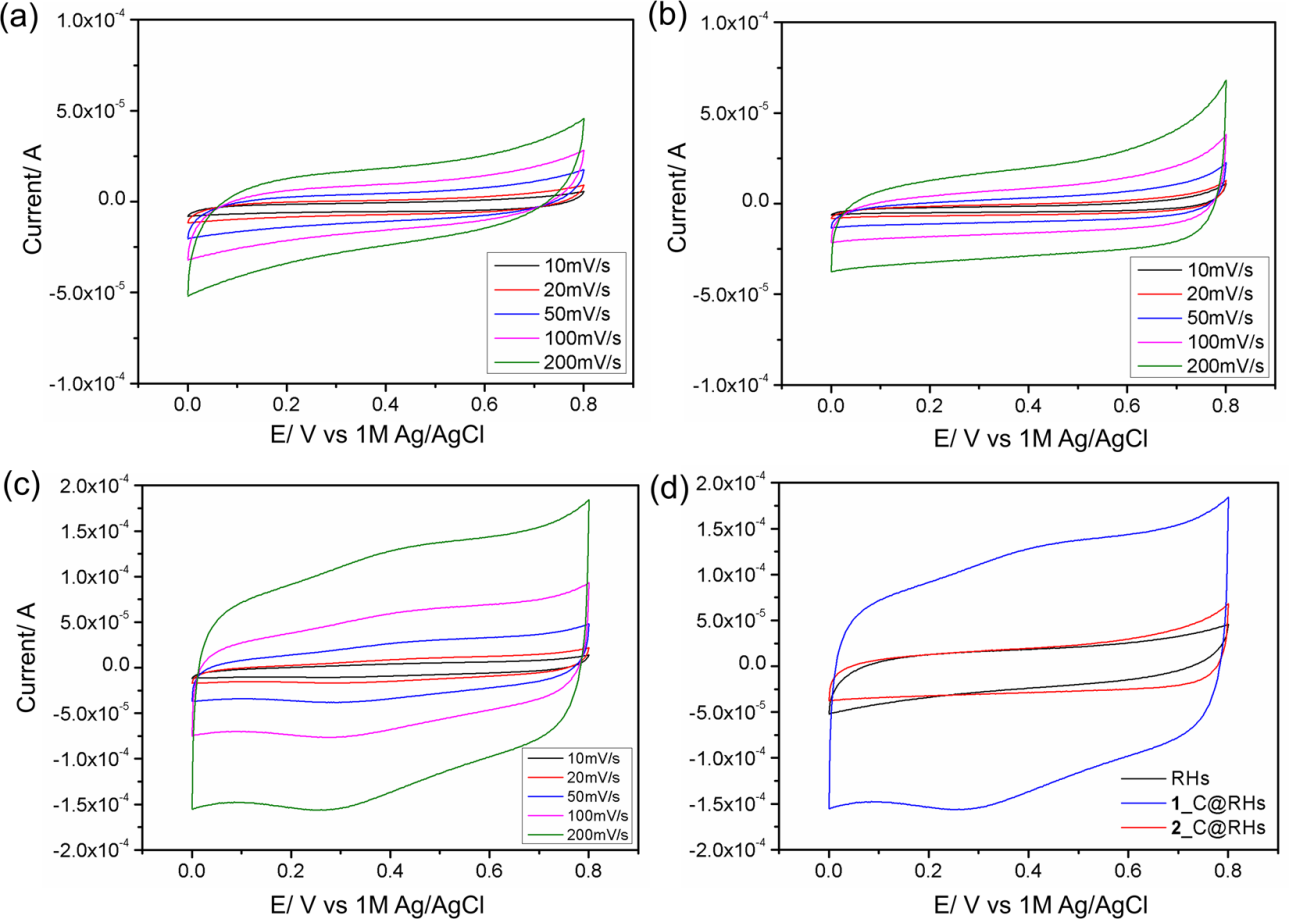
CV curves in 2 M KCl solution scanning from 0 to 0.8 V with scan rates of 10, 20, 50, 100 and 200 mV/s of (**a**) RHs, (**b**) **2**_C@RHs and (**c**) **1**_C@RHs, and (**d**) merged CV curves of all samples at scan rates of 200 mVs^−1^, Ag/AgCl (1 M KCl) electrode was used as reference.

The electrochemical properties of RHs, **1**_C@RHs and **2**_C@RHs were evaluated using a three-electrode configuration in 2 M KCl aqueous electrolyte solution. Figure 6 shows the CV curves in 2 M KCl solution at various scan rates ranging from 10 to 200 mV/s. At lower scan rates, a quasi-rectangular shape along the current/potential axis without obvious redox peaks is observed, which is typical for electric double-layer capacitance.

The CV curve of **1**_C@RHs is more symmetrical and exhibits larger currents than RH or **2**_C@RHs as a result of reversible pseudocapacitance which can be attributed to the changing of oxidation states of cobalt and manganese in CoMn_2_O_4_ as described by Eqs (1) and (2) below^57,58^.

1 $${{\rm{CoMn}}}_{{\rm{2}}}{\rm{O4}}+{{\rm{H}}}_{{\rm{2}}}{\rm{O}}+{{\rm{OH}}}^{-}\rightleftarrows \mathrm{Co}({\rm{O}})\mathrm{OH}+\mathrm{2Mn}({\rm{O}})\mathrm{OH}+{{\rm{e}}}^{-}$$

2 $$\mathrm{Mn}({\rm{O}})\mathrm{OH}+\mathrm{Co}({\rm{O}})\mathrm{OH}+{{\rm{2OH}}}^{-}\rightleftarrows {{\rm{MnO}}}_{{\rm{2}}}+{{\rm{CoO}}}_{{\rm{2}}}+{{\rm{2H}}}_{{\rm{2}}}{\rm{O}}+{{\rm{2e}}}^{-}$$

Similarly, MnO can be oxidized to Mn(O)OH and hence take part in the pseudocapacitance as described by Eq. (2). However, it should be noted that Co(0), which is present in the samples, will also be oxidized but is unlikely to contribute to the pseudocapacitive performance of the material.

The specific capacitance (F/g) of RHs, **1**_C@RHs and **2**_C@RHs at different scan rates are listed in Table 3. As can be seen, the capacitive performance for **1**_C@RHs maintains across all scan rates, suggesting ideal capacitive behavior, while the capacitances of RHs and **2**_C@RHs decrease with increasing scan rates. The decreasing trend is due to the slow formation of the electric double-layer as a result of the limited diffusion of the electrolyte ions within the pores^59,60^. The high electrochemical performance of **1**_C@RHs is proposed to be due to the higher loading amount of metal oxide and a larger mesopore volume which increases the pseudocapacitive and capacitive properties of the material respectively. In the former case, the increase in metal oxide uptake was a consequence of the smaller crystallite size of **1** allowing for facile uptake into the RH channels. In the latter case the higher mesopore volume allowed for increased surface area and hence greater EDLC.

**Table 3:** Specific capacitances of RHs, 1_C@RHs and 2_C@RHs at various scan rates.

Scan rate/ V·s^−1^	RHs/ Fg^−1^	1_C@RHs/ Fg^−1^	2_C@RHs/ Fg^−1^
10	13.1	30.3	9.4
50	8.2	28.4	7.1
100	6.7	30.7	6.8
200	5.6	32.8	6.8

To further corroborate the role of the RHs as a conductive host, **1** and **2** were carbonized to produce **1**_C and **2**_C respectively in the absence of RHs. The electrochemical performance of **1**_C and **2**_C was then measured using cyclic voltammetry in order to evaluate the capacitance of these materials with respect to the **1**_C@RHs and **2**_C@RHs. The results are shown in Table S1 and Fig. S10, and as expected, the performance of **1**_C and **2**_C was significantly lower than **1**_C@RHs and **2**_C@RHs respectively indicating that the RHs play a crucial role in enhancing the capacitance of the hybrid material, owing to its higher surface area and increased conductivity. Interestingly, the capacitance of **1**_C was found to be much greater than **2**_C which suggests that the morphology of **1** constituted an electrochemical advantage over **2** after carbonization which would contribute to the overall increased capacitance of **1**_C@RHs compared to **2**_C@RHs. In both cases, the specific capacitance of **1**_C and **2**_C decreased with increasing scan rate suggesting diffusion limitations of the electrolyte ions.

The electrochemical stability of **1**_C@RHs was investigated by galvanostatic charge-discharge (GCD) for 5000 cycles between 0 and 0.8 V at a current density of 0.5 A/g (see Fig. S11). The time taken for the GCD cycle to complete decreased from 138.9 s to 83.9 s going from the 200^th^ to the 5000^th^ cycle. The capacitance at the 200^th^ cycle was calculated to be 34.8 F/g in good agreement with the capacitance determined by CV in Table 3.

## Conclusions

Two different-sized bimetallic Co/Mn-MOFs were directly grown in the channels of pre-activated RHs, which is a conductive host, by changing the Mn(II) precursors. As confirmed by SEM and PXRD analysis, **1** has much smaller crystal size and grain size than those of **2** although both have an identical phase. The different crystal sizes of **1** and **2** were rationalized by considering the nucleophilic reactivity of the counter anions to the metal cations. Thus acetate used during the crystallization of **1** has a higher nucleophilic reactivity than chloride used to produce **2** in DMF, hence acetate is less readily solvated resulting in a smaller crystallite size. TGA data revealed that **1** with smaller crystal size has higher loading amount than **2** inside RHs since the larger crystal size of **2** can block the channels of the RHs and lead to lower loading capacities. The pyrolysis of **1**@RH and **2**@RH generates CoMn_2_O_4_, Co and MnO mixtures inside the RHs, which work as pseudocapacitive nanomaterials. Interestingly, the electrochemical performance of **1**_C@RHs is superior to that of **2**_C@RHs even though they have a similar surface area. The high capacitance of **1**_C@RHs across all scan rates is attributed to high loading amount of metal/metal oxide in RHs, higher mesopore volume, good electrical contact as well as the higher specific capacitance of **1**_C compared with **2**_C. The smaller crystallite size of **1** compared to **2** prevents the blockage of micrometer-scale channels of RHs, and increase the loading amount of **1** and the mesopore volume. Therefore, the crystal size control of MOFs in RHs is crucial to improve the electrochemical performance of pseudocapacitive nanomaterials. We believe that the impregnation of well-organized MOFs in biomass carbons could contribute to the design of novel energy storage devices with high performance.

## Experimental

### Materials

Manganese chloride (MnCl_2_), manganese acetate tetrahydrate (Mn(CH_3_CO_2_)_2_·4H_2_O) and cobalt nitrate hexahydrate (Co(NO_3_)_2_∙6H_2_O) were purchased from Sigma Aldrich. 2,5-Dihydroxyterephthalic acid (H_4_DOBDC) was purchased from TCI. All chemicals and solvents were commercially available and used without any further purification process. RHs which had been pre-activated under N_2_ atmosphere at 400 °C was obtained from Daewon Global System Integration.

### Synthesis of 1 and 2

Mn(CH_3_CO_2_)_2_ (0.040 g, 0.227 mmol)/Co(NO_3_)_2_∙6H_2_O (0.066 g, 0.227 mmol) for **1** or MnCl_2_ (0.030 g, 0.227 mmol)/Co(NO_3_)_2_∙6H_2_O (0.066 g, 0.227 mmol) for **2** with H_4_DOBDC (0.030 g, 0.15 mmol) were placed in a 20 mL vial and dissolved in a solution mixture of DMF (10 mL), ethanol (0.6 mL) and water (0.6 mL). The solution was heated at 130 °C for 16 h. After reaction, the solution was cooled to room temperature, and the product was collected on filter paper. Finally, the product was washed with DMF and methanol, and then dried under vacuum.

### Synthesis of 1_C@RHs and 2_C@RHs

One-pot crystallization of **1** and **2** in pre-activated RHs was performed using solvothermal reactions. Pre-activated RHs (30 mg) were added to the solutions of **1** and **2**, and the resulting mixture was heated up to 130 °C to crystallize the MOFs in the channels of pre-activated RHs. The products (**1**@RHs and **2**@RHs) were collected on filter paper. To synthesize metal/metal oxides in RHs (**1**_C@RHs and **2**_C@RHs), all samples were pyrolyzed at 700 °C for 6 h under nitrogen atmosphere.

### Characterization

The PXRD profiles were recorded using a Rigaku D/max 2500PC at 40 kV and 40 mA, employing Cu Kα radiation (λ = 1.5406 Å). SEM images were obtained by HITACHI S-4800. N_2_ adsorption isotherms were measured at 77 K using a BELSORP-MAX instrument. Samples were activated at 250 °C prior to measuring N_2_ adsorption isotherm. Transmission electron microscopy (TEM) analyses were performed using an aberration-corrected FEI Titan Themis 60–300 electron microscope operating at 80 kV and 300 kV, which is equipped with an energy-dispersive X-ray spectroscopy (EDS) detector (Oxford, X-Max 80). Both samples were dispersed in ethanol (1 mg/ml) by ultrasonic agitation and the solution was dropped on a carbon coated copper TEM-grid. For X-ray photoelectron spectroscopy, samples were analysed using a Thermo Scientific K-Alpha XPS instrument equipped with a microfocussed monochromated Al X-ray source. The source was operated at 12 keV and a 400 micron spot size was used. The analyser operates at a constant analyser energy (CAE) 200 eV for survey scans and 50 eV for detailed scans. Typically, survey and elemental region scans were recorded three and ten times respectively, with a 50 ms dwell time. Charge neutralization was applied using a combined low energy/ion flood source. The data acquisition and analysis was performed with Thermo Scientifics Avantage software. Normalized atomic percentages were determined from the peak areas of the elemental main peaks detected on the survey scan following a Smart background subtraction and application of Thermo sensitivity factors. Deconvolution of the Co2p_3/2_ region was carried out using CasaXPS software using Shirley background functions. The specific fitting parameters are described in the text.

### Electrochemical measurements

Electrochemical measurements were performed at room temperature (20 ± 2 °C) with an Autolab PGSTAT128N potentiostat (Metrohm Autolab, Utrecht, Netherlands) and NOVA software. The three-electrode system was used and consisted of a working electrode (glassy carbon), a counter electrode (Pt wire) and an Ag/AgCl (1 M KCl) reference electrode. The samples to be tested were first ball milled for 2 × 2 min at 200 rpm waiting 2 min in between, using a Fritsch premium line pulverisette 7 ball mill from Christison Particle Technologies. The glassy carbon electrode was then modified by drop casting 9 µL (2 mg/ml) of the ball milled suspension onto the electrode and allowing it to dry under ambient conditions. This was followed by the addition of 9 µL of Nafion 117 (from Aldrich, and used as received), and drying, as described previously.

Cyclic voltammetry (CV) was employed to evaluate the specific capacitance of each sample in 2 M KCl aqueous electrolyte using the following equation:

3 $${C}_{m}={\int }_{{E}_{1}}^{{E}_{2}}i(E)dE/2({E}_{2}-{E}_{1})m\nu $$

where ${C}_{m}$ is the specific capacitance (per gram) of the individual sample. ${\int }_{{E}_{1}}^{{E}_{2}}i(E)$ is the total voltammetric charge obtained by integration of the positive and negative sweep. E_1_ and E_2_ are the potential limits, m is the mass of the sample and *v* is the scan rate.

Galvanostatic charge-discharge (GCD) was performed by preparing the electrodes in the same way as described previously using CV. The electrodes were charged from 0 to 0.8 V for 5000 cycles at a constant current density of 0.5 A/g.

The specific capacitance (F/g) was evaluated using the formula:

4 $${{C}}_{{m}}=\frac{I({t}_{2}-{t}_{1})}{m({E}_{2}-{E}_{1})}$$

where ${C}_{m}$ is the specific capacitance (per gram) of the individual sample; I is the current density (0.5 A/g), t_2_-t_1_ is the discharge time, m is the mass of the sample and E_2_-E_1_ is the potential window.

All chemicals were purchased from Sigma-Aldrich and were of A.C.S. reagent grade. Aqueous solutions were prepared with Milli-Q water (>18 MΩ cm).

## Supplementary information

Electronic Supplementary Inforamtion
