# Supplementary material for: Facile One-Pot Synthesis of Bimetallic Co/Mn-MOFs@Rice Husks, and its Carbonization for Supercapacitor Electrodes
Source: Sci Rep. 2019 Jun 20;9:8984. doi: 10.1038/s41598-019-45169-0 (PMC6586648; doi:10.1038/s41598-019-45169-0)
Supplement: Supplementary file 1 — Electronic Supplementary Inforamtion [file 41598_2019_45169_MOESM1_ESM.docx]

**Facile One-Pot Synthesis of Bimetallic Co/Mn-MOFs@Rice Husks, and its Carbonization for Supercapacitor Electrodes**

Hyunuk Kim,^†,⊥,^* Muhammad Sohail,^†,⊥^ Chenbo Wang,^‡^ Martin Rosillo-Lopez,^‡^ Kangkyun Baek,^#^ Jaehyoung Koo,^#^ Myung Won Seo,^§^ Seyoung Kim,^†^ John S Foord,^‡,^* Seong Ok Han,^†,^*

^†^Energy Materials Laboratory and ^§^Green Fuel Laboratory, Korea Institute of Energy Research, 152 Gajeong-ro, Yuseong-gu, Daejeon 34129, Republic of Korea

^⊥^Advanced Energy and Technology, University of Science and Technology (UST), Daejeon, 34113, Republic of Korea

^#^Center Center for Self-assembly and Complexity, Institute for Basic Science, 77 Cheongam-ro, Nam-gu Pohang 37673, Republic of Korea

^‡^Department of Chemistry, University of Oxford, South Parks Road, Oxford, OX1 3TA, United Kingdom


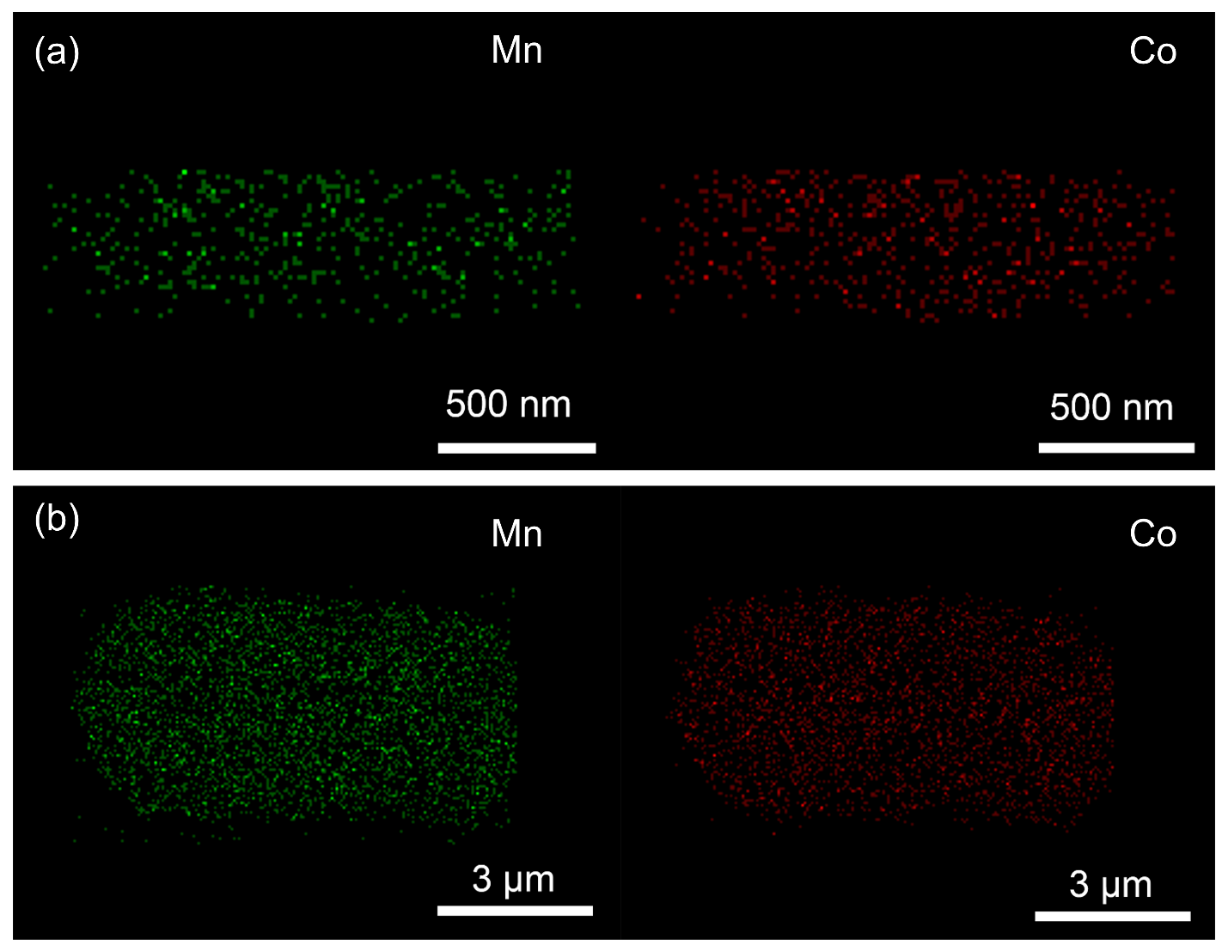


Figure S1. SEM-EDX mapping of single-crystals for **1** (a) and **2** (b)


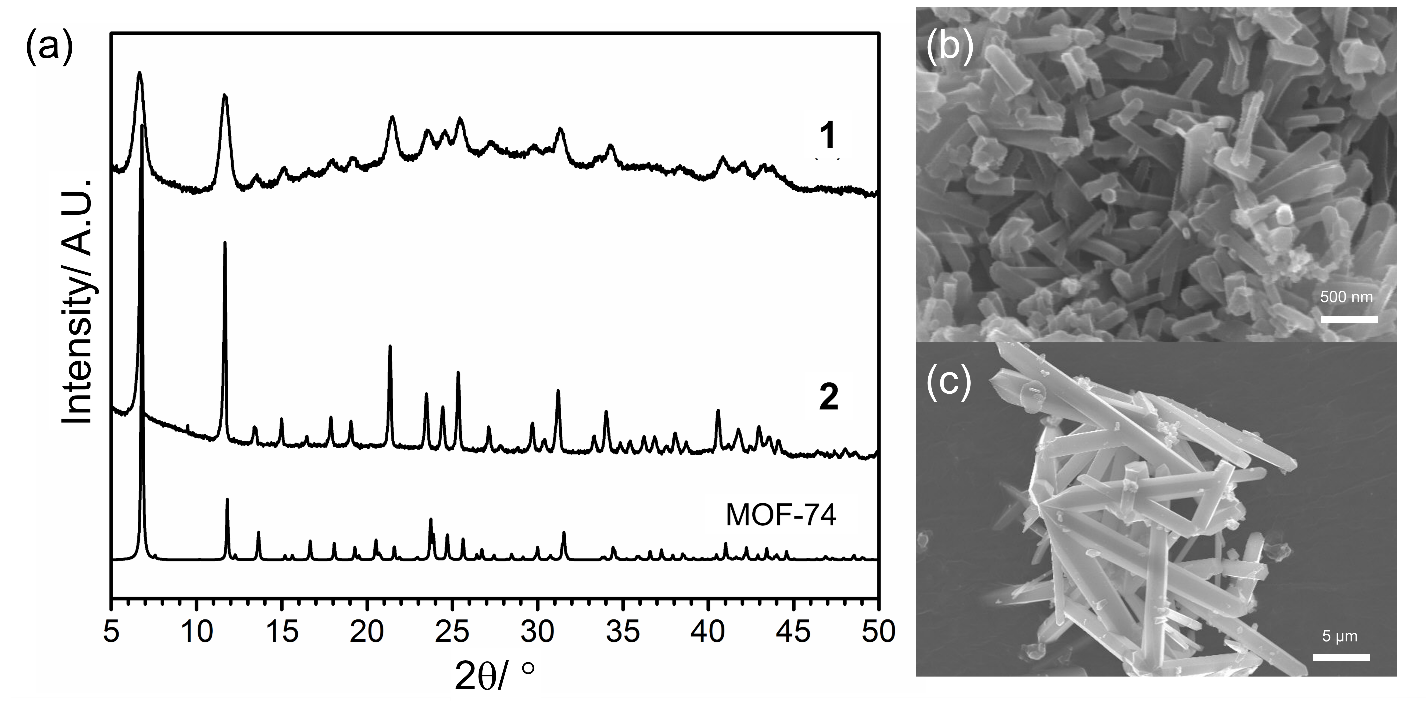


Figure S2. (a) Powder X-ray diffraction (PXRD) profiles of **1** and **2**, SEM images of **1** (b) and **2** (c)


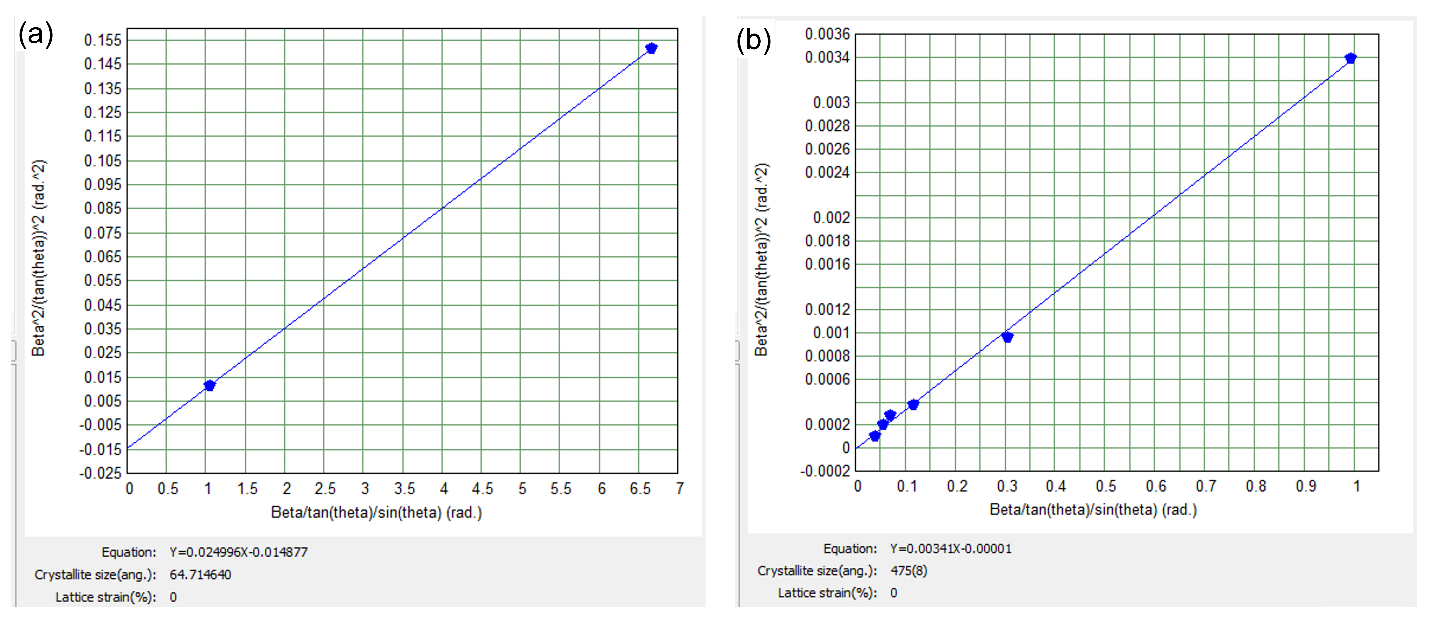


Figure S3. Crystallite sizes of **1** (a) and **2** (b) estimated by the Halder-Wagner Method


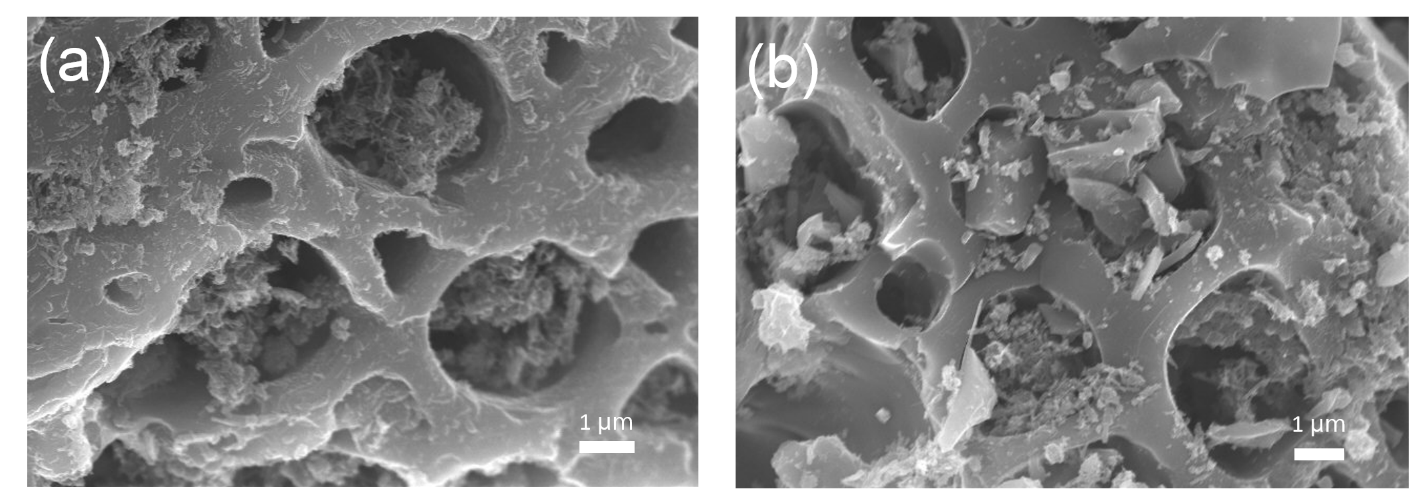


Figure S4. SEM images of (a) **1**@RHs, (c) **2**@RHs


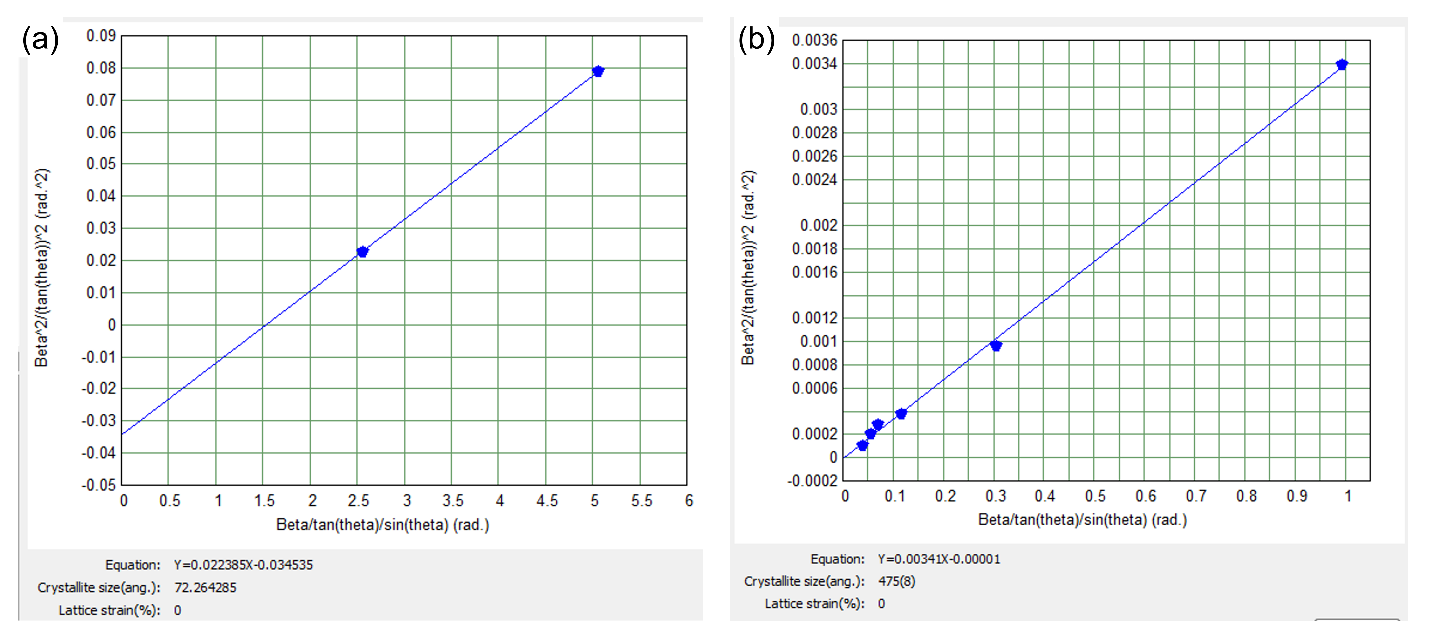


Figure S5. Crystallite sizes of **1**@RHs (a) and **2**@RHs (b) estimated by the Halder-Wagner Method


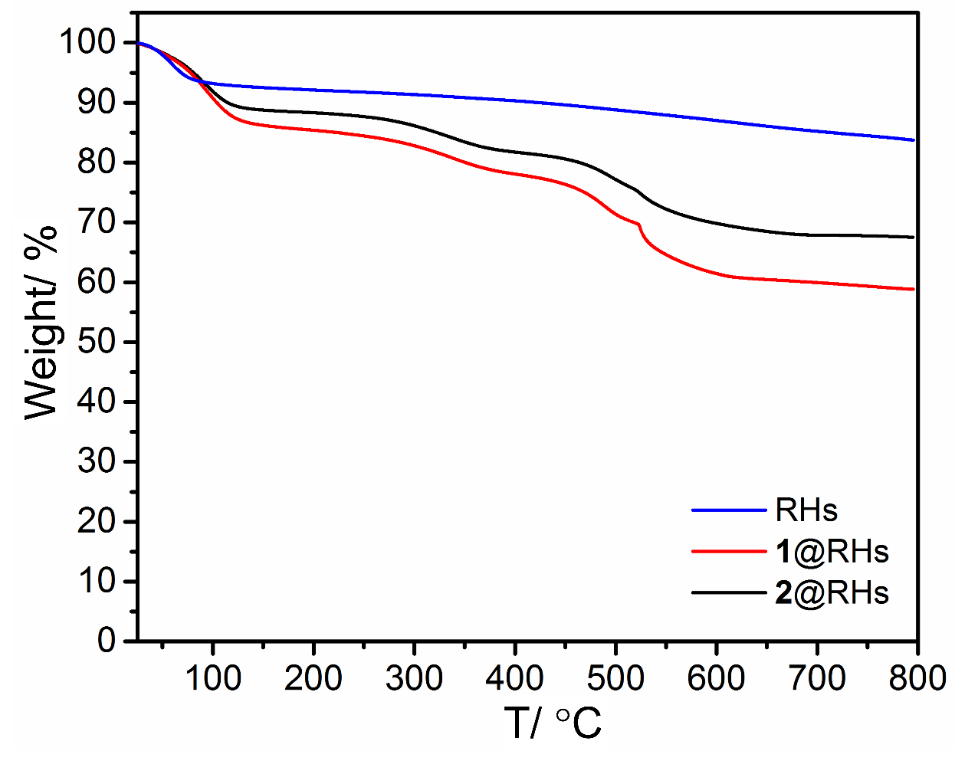


Figure S6. TGA data of RHs, **1**@RHs and **2**@RHs


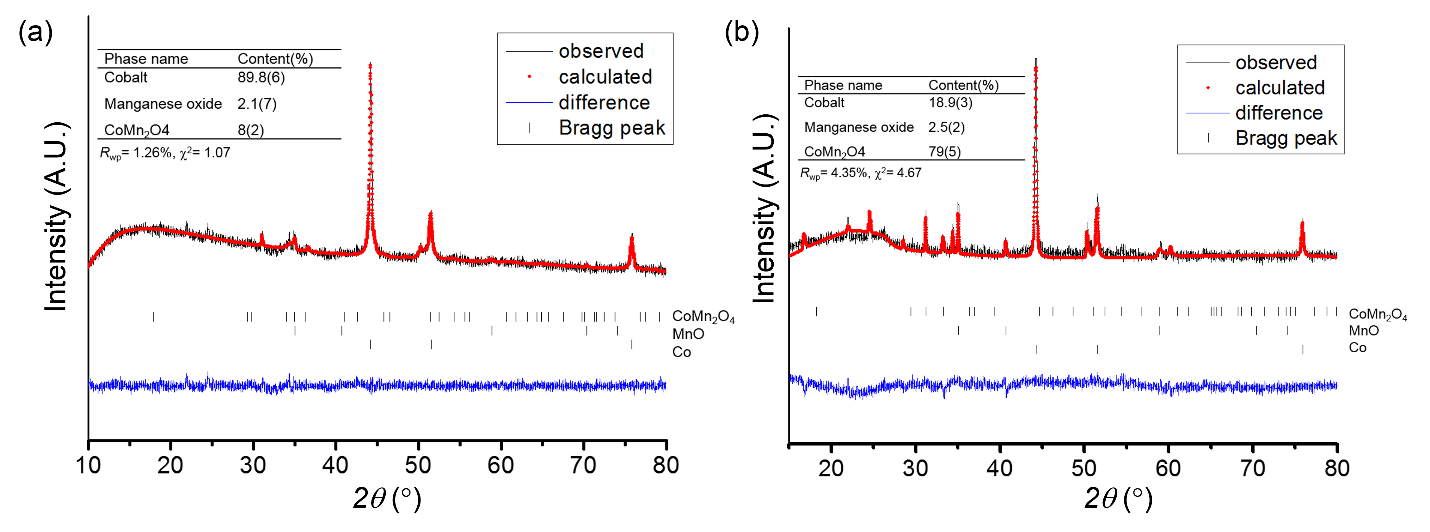


Figure S7. Rietveld refined XRD patterns for (a) **1**_C@RHs (R_wp_=1.26%) and (b) **2**_C@RHs (R_wp_=4.35%) with the structural models for Co, MnO, and CoMn_2_O_4_


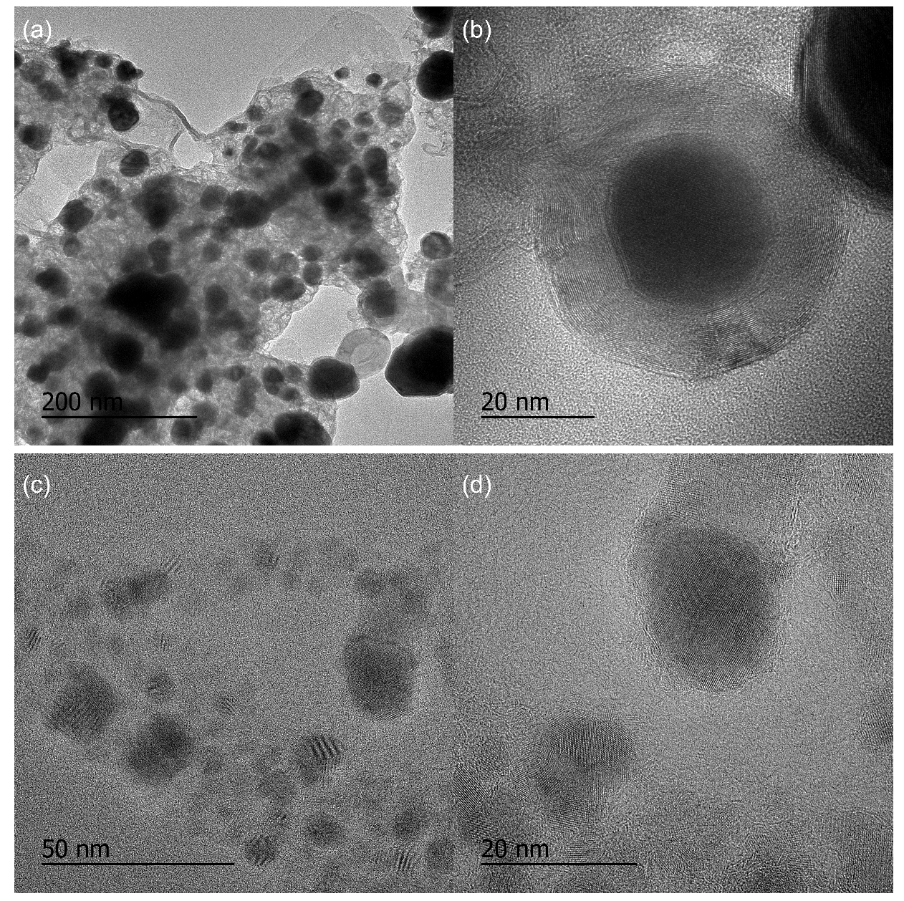


Figure S8. HRTEM images of **1**_C@RHs (a,b) and **2**_C@RHs (c,d)


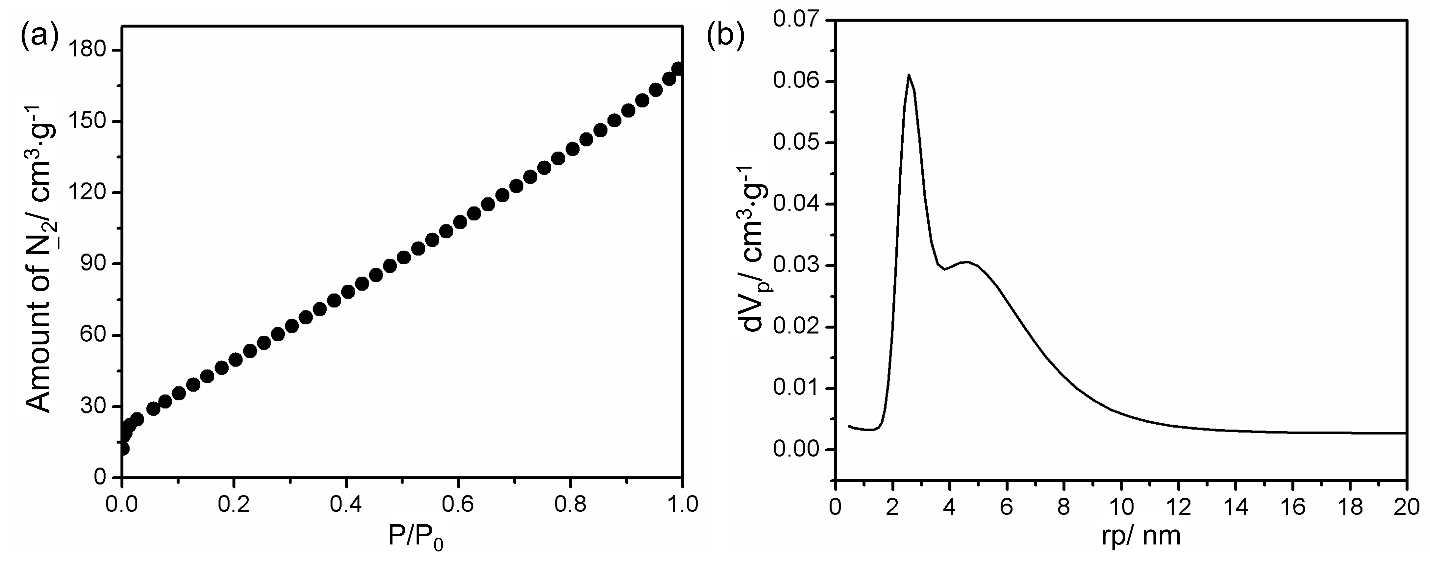


Figure S9. (a) N_2_ adsorption isotherm and (b) pore size distribution of RHs by NLDFT

Table S1. Specific capacitances of RHs, 1_C and 2_C at various scan rates.

| Scan rate/ V·s^-1^ | **1_C**/ Fg^-1^ | **2_C**/ Fg^-1^ |
| --- | --- | --- |
| 10 | 18.2 | 5.9 |
| 20 | 13.6 | 4.2 |
| 50 | 10.3 | 3.6 |
| 100 | 8.5 | 3.1 |
| 200 | 6.6 | 2.4 |

Figure S10. CV curves of (a) **1**_C and (b) **2**_C in 2 M KCl aqueous solution scanning from 0 to 0.8 V and back, vs Ag/AgCl (1 M KCl) at scan rates of 10, 20, 50, 100 and 200 mV/s.

Figure S11. Galvanostatic charge-discharge (GCD) curves at 0.5 A g ^-1^ of **1**_C@RHs, showing every 200^th^ cycle up to 5000 cycles.
